# Primary Health Care Management Effectiveness as a Driver of Family Planning Service Readiness: A Cross-Sectional Analysis in Central Mozambique

**DOI:** 10.9745/GHSP-D-21-00706

**Published:** 2022-09-15

**Authors:** Stephen Pope, Orvalho Augusto, Quinhas Fernandes, Sarah Gimbel, Isaías Ramiro, Dorlim Uetela, Stélio Tembe, Meredith Kimball, Mélia Manaca, C. Leigh Anderson, Sérgio Chicumbe, Kenneth Sherr

**Affiliations:** a Department of Global Health, University of Washington, Seattle, WA, USA.; b Faculty of Medicine, Eduardo Mondlane School of Medicine, Maputo, Mozambique.; c Ministry of Health, Maputo, Mozambique.; d Department of Family and Child Nursing, University of Washington, Seattle, WA, USA.; e Comité para Saúde de Moçambique, Maputo, Mozambique.; f Daniel J Evans School of Public Policy & Governance, University of Washington, Seattle, WA, USA.; g Instituto Nacional de Saúde de Moçambique, Maputo, Mozambique.; h Department of Industrial & Systems Engineering, University of Washington, Seattle, WA, USA.; i Department of Epidemiology, University of Washington, Seattle, WA, USA.

## Abstract

**Introduction:**

The effectiveness of facility-level management is an important determinant of primary health care (PHC) reach and quality; however, the nature of the relationship between facility-level management and health system effectiveness lacks sufficient empirical grounding. We describe the association between management effectiveness and facility readiness to provide family planning services in central Mozambique.

**Methods:**

We linked data from the Ministry of Health’s 2018 Service Availability and Readiness Assessment and a second 2018 health facility survey that included the World Bank’s Service Delivery Indicators management module. Our analysis focused on 68 public sector PHC facilities in Manica, Sofala, Tete, and Zambézia provinces in which the 2 surveys overlapped. We used logistic quantile regression to model associations between management strength and family planning service readiness.

**Results:**

Of the 68 facility managers, 47 (69.1%) were first-time managers and (18) 26.5% had received formal management training. Managers indicated that 63.6% of their time was spent on management responsibilities, 63.2% of their employees had received a performance review in the year preceding the survey, and 12.5% of employee incentives were linked to performance evaluations. Adjusting for facility type and distance to the provincial capital, facility management effectiveness, and urban location were significantly associated with higher levels of readiness for family planning service delivery.

**Conclusions:**

We found that a higher degree of management effectiveness is independently associated with an increased likelihood of improved family planning service readiness. Furthermore, we describe barriers to effective PHC service management, including managers lacking formal training and spending a significant amount of time on nonmanagerial duties. Strengthening management capacity and reinforcing management practices at the PHC level are needed to improve health system readiness and outputs, which is essential for achieving global Sustainable Development Goals and universal health coverage targets.

## Abstract (precis)

We found higher levels of management effectiveness in primary health care facilities to be independently associated with an increased likelihood of improved family planning service readiness in central Mozambique. Strengthening management capabilities and reinforcing management roles at the primary health care level may improve health system readiness and provision of quality family planning services.

## Teaser

**Key Findings:**

- About 74% of primary health care (PHC) facility managers surveyed did not receive any kind of formal management training, identifying a gap in building management capacity.
- Facility managers did not conduct routine staff performance reviews, making it challenging for managers to identify areas for performance improvements.
- Analysis of facilities included in this assessment revealed significant gaps in the quality and frequency of supervisory visits.

**Key Implications:**

- Valid and feasible indicators that are sensitive to PHC management practices and service readiness could improve the comparability and generalizability of PHC implementation research.
- Investments in management quality improvement and focused efforts to increase performance management practices in PHC settings are necessary for policy makers to support improved family planning service readiness.

Resumo em português no final do artigo.

## INTRODUCTION

Availability of quality primary health care (PHC) and reduction in unmet demand for modern contraception are recognized as important milestones toward achieving the Sustainable Development Goals and universal health coverage.^1^^–^^3^ PHC facilities are a key access point for family planning (FP) services in many low-resource settings: in Mozambique, 94% of health facilities—of which 96% are PHC facilities—provide FP services for adults.^4^ However, a recent national study found that only about one-quarter of health facilities met the readiness criteria to provide quality FP services, highlighting a major barrier to achieving quality health outcomes.^4^ Although multiple health system building blocks directly influence PHC outcomes, facility-level organizational management may be among the most influential in improving service delivery and ultimately health outcomes.^5^ Previous literature from high- and middle-income countries suggests that effective management practices are associated with improved health facility performance, as evidenced by reductions in mortality rates in emergency room settings and strengthened financial performance.^6^^,^^7^ However, there is limited evidence on such associations within low-income countries.^8^

Management competencies such as strategic thinking, human resource management, financial and operational management, performance management, governance and leadership, and community engagement are necessary to maximize efficiencies to meet the health needs of populations in resource-constrained settings.^8^ Previous research about PHC in Mozambique describes the management environment and highlights some of the challenges that limit service coverage and quality.^9^ Notably, district management teams face challenges with regard to resource allocation, planning, financial management, and data-driven decision making.^9^ These competencies are broadly recognized as critical for improving PHC service delivery and health outcomes.^5^^,^^8^^,^^10^ However, research examining the strength of organizational management and its role in improving health system effectiveness is limited by both a lack of standardized tools to quantify management strength and a lack of research on its association with health system performance, particularly in low- and middle-income settings.^5^^,^^8^^,^^10^^,^^11^

Although varied in their scope and rigor, earlier studies found that management-strengthening interventions can lead to performance enhancements, such as improved medical records, clinical guideline adherence, and reduced waiting times.^8^ More recently, studies have described a direct relationship between effective facility-level management and health systems outputs.^5^^,^^8^^,^^12^ A cross-sectional study of 142 health facilities across Ghana found that those with higher management scores (assessed using the World Management Survey) had significant positive associations with the practice of integrating FP into maternal and child health and HIV services, the types of FP methods provided, and availability of essential equipment.^13^ Another cross-sectional study including 221 health facilities across 4 regions of Ethiopia described significant positive associations between health facilities with higher management scores (assessed using Ethiopia’s Woreda Management Standards survey) and 5 health system performance indicators, including antenatal care coverage, contraceptive acceptance, skilled birth attendance, childhood immunization, and essential drug availability.^14^ While these studies are important contributions to the literature about the role of management effectiveness and health systems performance, there is limited research investigating the associations between facility-level management practices and service readiness at the PHC level, including readiness to provide FP services. The lack of such research limits our efforts to understand the importance of management in strengthening health systems; it also complicates evaluations designed to generate an evidence base about approaches to improve management and strengthen health systems.

There is limited research investigating the associations between facility-level management practices and service readiness at the PHC level.

Our objective was to address the evidence gap on the association between PHC management effectiveness and readiness to provide adult FP services in central Mozambique. Our specific research objectives were: (1) to characterize management practices at the PHC level; and (2) to describe associations between PHC management strength and facility readiness to provide FP services.

## METHODS

### Setting

Use of PHC services is high across Mozambique, with first antenatal care visit coverage exceeding 90% and high levels of use of basic preventive and curative maternal, newborn, and child health services.^9^^,^^15^ High PHC use is driven by the Mozambique National Health Service’s strategy to rapidly expand PHC services through a network of health facilities, which increased from 755 to 1,300 facilities (48%) between 2007 and 2015.^16^ PHC facilities are classified by type, including rural health center types I and II and urban health center types A, B, and C; and facility type determines staffing and service availability patterns.^17^^,^^18^ For example, rural health centers type I and type II were originally designed to serve rural communities with populations of 7,500–20,000 (type I) and 16,000–35,000 (type II), with minimum staffing requirements of 4 (type I) and 13–16 (type II) health workers.^17^ Conversely, urban health centers types A, B, and C serve more densely populated areas.^17^ Type C urban health centers provide similar services as rural type I, and urban type B and rural type II provide similar services.^17^ Type A urban health centers provide similar services to type B, but with larger catchment areas and staffing requirements.^17^ PHC facilities are overseen by district management teams that report to provincial health directorates, which are key organizational units for managing, coordinating, and scaling PHC services across Mozambique’s 11 provinces.^9^ The provincial directorates and district management teams are deeply engaged in coordinating the implementation of the National Family Planning Program at the PHC facility level, which is a critical component of the Government of Mozambique’s strategy to improve health outcomes.^4^^,^^18^

### Data Sources

We drew our baseline data from a 5-year mixed-methods evaluation of the Integrated District Evidence-to-Action program to improve maternal, newborn, and child health (IDEAs) in central Mozambique. IDEAs uses a modified audit-and-feedback implementation strategy to improve the coverage and quality of a package of existing evidence-based interventions, targeting major causes of neonatal mortality in 12 districts and 72 health facilities in Manica and Sofala provinces (Figure). We sourced data for our analysis from 2 facility-level surveys conducted in 2018, namely the Mozambique National Health Institute’s Service Availability and Readiness Assessment (SARA) survey and the baseline IDEAs facility survey (conducted in intervention and control facilities in Manica, Sofala, Tete, and Zambézia provinces).^19^

**FIGURE:**
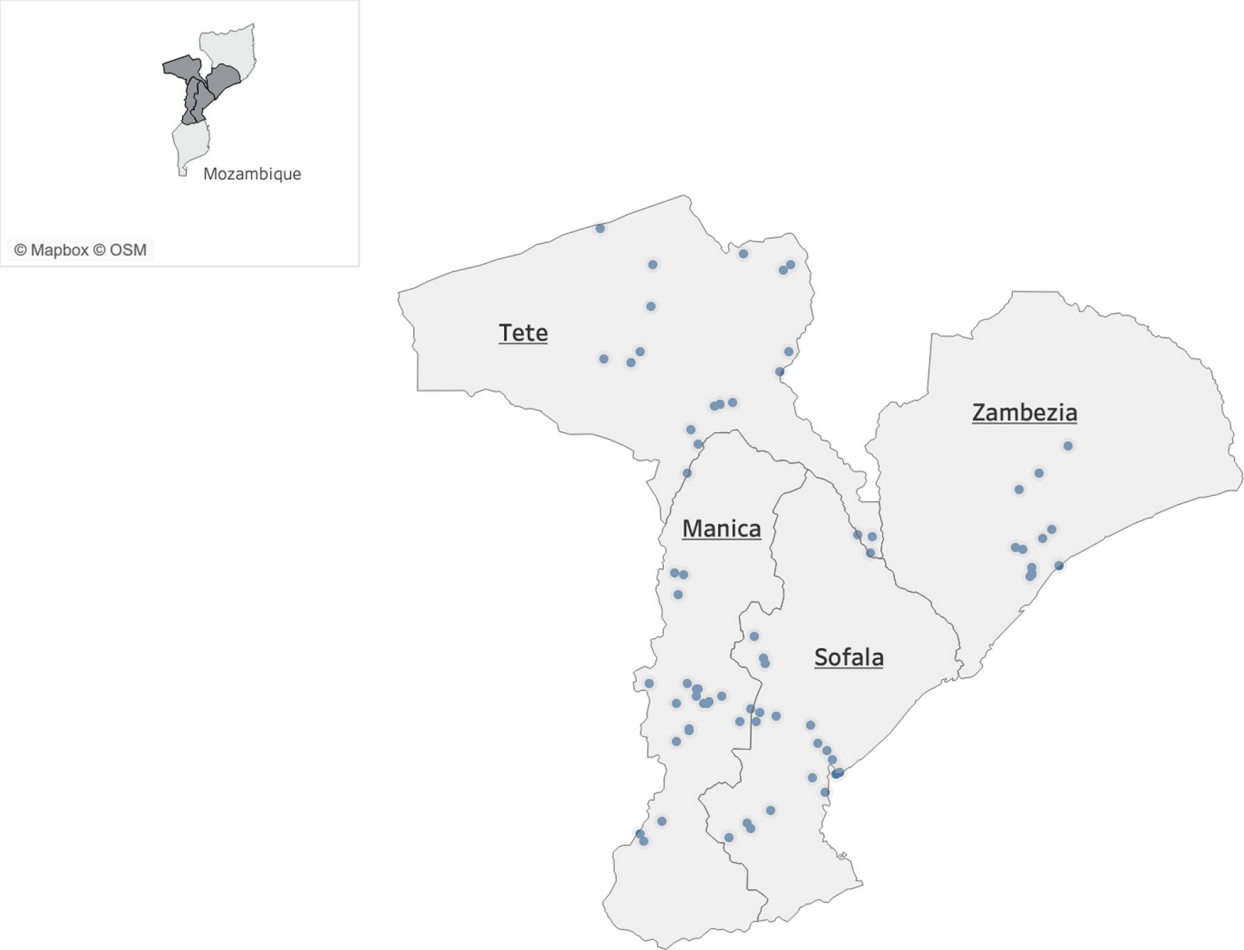
Distribution of Integrated District Evidence-to-Action Strategy Health Facilities in Central Mozambique (N=72)

#### SARA

SARA is a World Health Organization (WHO) standardized facility-level survey that contains a set of indicators designed to measure service availability, general service readiness, and service-specific readiness for a range of PHC service areas.^20^ Service-specific readiness refers to assessing the ability of a health facility to provide a specified service, such as FP, by documenting the availability of a set of tracer inputs including trained staff, guidelines, equipment, diagnostic capacity, and medicines and commodities.^20^ The FP readiness module assesses indicators about the prescription and provision of modern methods of contraception, FP guidelines and checklists, trained staff, current commodity stock, and 3-month commodity stock-out records.^19^ Because the Mozambique 2018 SARA survey was conducted by the Mozambique National Heath Institute (Instituto Nacional de Saúde) over a similar time frame as our project-specific facility survey, the SARA survey database was integrated with our baseline facility survey (including all SARA data from the 72 health facilities in our sample).^19^ For our analysis, we included only indicators related to the FP service-specific readiness assessment (Supplement 1).

#### IDEAs Survey

The IDEAs Study team developed the IDEAs survey to populate the evaluation framework for the IDEAs intervention, specifically focusing on the intervention’s impact on improving structural readiness, process quality, and management effectiveness. The survey instrument compiled relevant modules from multiple existing surveys. To quantify facility-level management effectiveness, the IDEAs facility survey incorporated a selection of measures from the Health Service Delivery Indicators (SDI) survey, which had been previously adapted and applied in Mozambique in 2014.^21^ The SDI initiative—a partnership of the World Bank, African Economic Research Consortium, and African Development Bank—aims to assess service delivery quality and link with other research studies to capture inputs in policy and institutional environments as well as health outcomes.^21^ The health SDI survey has 46 questions about health facility management divided across the following themes: roles and responsibilities in the facility, supervision from organizational leadership, time utilization, leadership, management practices, and management experience.^22^

For the IDEAs survey, a research team visited a sample of health facilities from the 12 intervention districts and 12 matched control districts. They collected data via structured interviews with facility managers and facility staff to assess management effectiveness and organizational climate, use of vignettes with facility staff to assess knowledge of clinical guidelines, and direct observation of clinical interactions to assess adherence to national guidelines. The management effectiveness module included indicators from the health SDI management and governance survey (Supplement 2) designed to document management effectiveness, as well as questions for facility managers to describe management procedures in their respective PHC facilities. For our study, we only analyzed the data collected from PHC facility managers.

#### Survey Sample

A total of 72 PHC facilities in central Mozambique were included in the IDEAs health facility survey, including 36 intervention facilities split evenly across 12 intervention districts in Manica and Sofala provinces, and a control group of 36 nonintervention facilities split evenly across 12 matched nonintervention districts in Tete and Zambézia provinces. Control districts were selected in a pairwise fashion matched to each intervention district based on population size, location (rural or urban), distance from the district capital to the provincial capital, and the facility network (both number and distribution of facility types).

Within each intervention district and control district, we selected 3 health facilities for data collection using procedures previously applied for similar facility-level surveys in the study area.^23^ First, the highest-volume facility (defined by the number of first antenatal care visits in 2017) was selected in each district. Given that most districts in Mozambique have 1 large facility with more staff and a more robust range of available PHC services, this approach ensured that we included the facility providing the bulk of PHC services in each district. Second, we randomly selected 2 additional facilities in each district from the remaining health facilities with at least 200 institutional births per year. By restricting study facilities to those with at least 200 institutional deliveries per year, we excluded very small health facilities, which have a relatively smaller impact on population-level health outcomes.

#### Source of Subjects

All facility directors and health facility employees of the Mozambique National Health Service in the study were eligible to participate in the research. Facility staff who were not supported by the National Health Service and those without clinical health training (e.g., administrative staff without any clinical role in the facility) were excluded from the study.

#### Sample Size and Response Rates

Of the 72 health facilities included in the survey, facility managers (directors of each facility) from 70 facilities responded. We excluded 4 of the 72 facilities from our analysis: 2 rural facilities whose facility managers were unavailable during the data collection period; and 2 additional facilities (1 rural type II facility and 1 urban type B facility), for which we were unable to obtain SARA data. The final sample, therefore, included a total of 68 PHC facilities and 68 facility managers.

#### Data Collection

The IDEAs study data were collected over a 6-month period beginning in the second semester of 2018. Study personnel were trained in study procedures and research ethics and organized into 1 team that sequentially visited each study facility. The team collected and managed study data using REDCap (Research Electronic Data Capture) electronic data capture tools hosted at the University of Washington.^23^

### Analysis

We used standard descriptive statistics to describe the health facilities’ location (rural or urban), facility type, distance from the provincial health directorate, and staffing. In addition, we used descriptive statistics to characterize the health facility managers surveyed in terms of the number of years of management experience, whether they were first-time managers, and whether they had received any management training.

#### Management Effectiveness

The main explanatory variable in FP service readiness at the PHC facility level was management effectiveness. We categorized the 17 management component indicators from the IDEAs management survey into 3 management practice domains: management practices, external supervision, and community engagement. All indicators were either binary or ordinal measures. Each indicator was then scored on a scale from 0 (lowest) to 1 (highest). We created a composite management score based on the average score for each of the 3 domains (Supplement 3). We also analyzed 2 additional variables, namely the number of years of management experience each facility manager had, and whether the facility manager was a first-time manager.

#### Facility Readiness

The main outcome variable was facility readiness for FP services at the PHC facility level. The 26 component indicators from the SARA FP readiness module are organized into 5 domains: provision and prescription, guidelines and checklists, trained staff, current stock, and stock-outs. All indicators were either binary or ordinal measures. Each indicator was then scored on a scale from 0 (lowest) to 1 (highest). We created a composite readiness score based on the average score for each of the 5 readiness domains (Supplement 4).

#### Regression Analysis

We used robust logistic linear quantile regression to analyze the association between management-effectiveness scores and facility-readiness scores. Logistic quantile regression models the conditional quantile of the outcome rather than the conditional mean.^24^^,^^25^ We chose this model, rather than a generalized linear regression model (which models the mean of the outcome), because it is particularly useful for skewed data and for bounded outcomes.^25^^,^^26^ We based our model on the median quantile of the overall FP readiness score. Using this approach, the exponentiated coefficients are interpreted as a change in the odds ratios for a score above the median quantile of the overall readiness score given a 1-unit change in the explanatory variable. For each explanatory variable (overall management score, years of management experience, first-time manager), we built 3 models: Model A, unadjusted; Model B, adjusted for health facility type (rural health center, urban health center, or health center adjoining a hospital); and Model C, adjusted for facility type and distance to the provincial health directorate.

We used RStudio version 1.1.383 (Rstudio, Boston, MA) to conduct all analyses; associations were evaluated for statistical significance at α=.05 using 2-tailed tests.

### Ethics Approval

Novel data collection procedures were approved by the institutional review board of the University of Washington (IRB#STUDY00003926), Mozambique’s National Bioethics Committee for Health (Comité Nacional de Bioética para a Saúde) of Mozambique (IRB00002657), and Ministry of Health—after endorsement from the provincial health directorates in Manica, Sofala, Tete, and Zambézia. We conducted interviews after obtaining written informed consent from key informants. Data collection used individual alphanumeric codes that protected the identity of each key informant.

## RESULTS

### Description of PHC Facilities and Facility Managers

A total of 72 health facilities participated in the IDEAs survey. After excluding 4 facilities due to lack of explanatory or outcome data, we included 68 health facilities in the final analysis, representing 94.4% of the IDEAs survey sample. Among the 68 facilities, the majority (n=54, 79.4%) of health facilities surveyed were in rural areas and located an average of 125.4 km from a provincial health directorate (Table 1). Rural health centers (types I and II) made up three-quarters (n=51, 75.0%) of the sample, followed by urban health centers (n=14, 20.1%) and health centers adjoining secondary-level hospitals (n=3, 4.4%), all of which offer FP services.

**TABLE 1.** Characteristics of Health Facilities and Facility Managers Analyzed, Central Mozambique

		**Median (IQR)**
**Health facilities (N=68), mean (SD)**		
Number of total staff	30.2 (38.4)	9.0 (4.0, 51.5)
Number of clinical staff	20.7 (26.2)	5.0 (3.0, 34.3)
Number of pharmacy staff	2.1 (2.8)	1.0 (0.0, 4.0)
Number of laboratory staff	2.0 (3.7)	0.0 (0.0, 3.0)
Number of administrative staff	5.3 (10.7)	1.0 (0.0, 3.3)
Distance to provincial health directorate in km	125.4 (109.9)	104.0 (29.75, 187.5)
**Rural facilities, No. (%)**	54 (79.4)	
Rural health center Type I	9 (13.2)	
Rural health center Type II	42 (61.8)	
Health center adjoining secondary-level hospital	3 (4.4)	
**Urban facilities, No. (%)**	14 (20.1)	
Urban health center type A	4 (5.9)	
Urban health center type B	8 (11.8)	
Urban health center type C	2 (2.9)	
**Facility managers (N=68)**		
Years of experience in the health sector	8.4 (8.4)	5.0 (3.0, 8.7)
Years of experience as a facility manager	2.5 (2.1)	2.0 (1.0, 3.0)
First-time health facility managers, No. (%)	47 (69.1)	
Received formal management training	18 (26.5)	

Abbreviations: IQR, interquartile range; SD, standard deviation.

Over two-thirds (n=47, 69.1%) of the 68 facility managers surveyed were first-time managers; one-quarter of the managers had less than 1 year of experience as a manager; and three-quarters had less than 3 years of experience as managers. Approximately one-quarter (n=18, 26.5%) of managers had ever received any type of formal health management training (Table 1).

The average overall management score was 0.59 (standard deviation [SD] 0.17), with moderate variation across the 3 management domains (Table 2). The “management practices” domain scored the highest (mean 0.63, SD 0.18), but there was substantial variation between the measures. Most managers performed essential management practices such as recording staff absences (mean 0.90, SD 0.30) and requesting monthly medication refills during the previous 6 months (mean 0.88, SD 0.32). Duties related to performance management, such as conducting performance reviews (mean 0.63, SD 0.49) and linking staff salaries and incentives to work performance (mean 0.12, SD 0.33), were not commonly practiced. On average, facility managers spent more than a third of their time on nonmanagerial tasks such as clinical duties.

**TABLE 2.** Management Effectiveness Scores Among Study Facilities^a^ Central Mozambique, (N=68)

	**Mean (SD)**
Overall management effectiveness score	0.59 (0.17)
Management practices domain score	0.63 (0.18)
Proportion of time managers spent on managerial duties	0.63 (0.16)
Facility keeps records of staff absences	0.90 (0.31)
Facility conducted performance reviews with employees in past 12 months	0.63 (0.49)
Facility linked work performance to employee salaries and incentives	0.12 (0.33)
Facility requested medication once per month in the past 12 months	0.88 (0.33)
External supervision domain score	0.54 (0.29)
Facility received 6 external supervisory/technical visits in past 6 months^b^	0.65 (0.42)
External supervisor used a control or verification list during most recent visit	0.66 (0.48)
External supervisor observed consultations during most recent visit	0.59 (0.50)
External supervisor observed staff attendance logs during most recent visit	0.53 (0.50)
External supervisor observed stocks of medications during most recent visit	0.72 (0.45)
External supervisor observed financial registries during most recent visit	0.31 (0.47)
Facility staff received an evaluation summary from external supervisor during most recent visit	0.30 (0.43)
Community engagement domain score	0.59 (0.27)
Facility community health committee met monthly during the past 12 months^c^	0.42 (0.38)
Facility collects patient opinions through a formal mechanism	0.75 (0.39)
Facility made management changes based on patient opinions in past 6 months	0.62 (0.49)

a Overall management effectiveness domain scores are averages of individual component indicators on a scale of 0 (lowest) to 1 (highest). All individual component indicators are binary from 0 (No) to 1 (Yes) unless otherwise specified.

b Fewer than 3 visits (0); 3 or 4 visits (0.5); 5 or more visits (1).

c Fewer than 3 meetings (0); 3 to 5 meetings (0.25); 6 to 11 meetings (0.5); 12 or more meetings (1).

Most managers performed essential management practices such as recording staff absences and requesting medication refills.

The “external supervision” domain scored the lowest (mean 0.54, SD 0.29), with just over half (n=36) of facility managers reporting that they received a monthly supervisory visit during the previous 6 months. Similar to facility managers, external supervisors generally did not conduct evaluation summaries for facility staff during their visit (mean 0.30, SD 0.43).

There was substantial variation between the indicators in the “community engagement” domain, which had a mid-level score (mean 0.59, SD 0.27). Although 59 facility managers reported having a community health committee, only 17 confirmed having monthly meetings during the past 12 months (mean 0.42, SD 0.38). Although many facility managers reported collecting patient feedback through a formal mechanism (mean 0.75, SD 0.39), fewer managers implemented management changes based on patient feedback (mean 0.62, SD 0.49).

### FP Facility Readiness

The average overall FP facility readiness score was 0.69 (SD 0.20) (Table 3). The “guidelines and checklists” domain (mean 0.74, SD 0.38) and the “trained staff” domain (mean 0.72, SD 0.45) scored the highest among the sample facilities. The “provision and prescription” domain scored the lowest (mean 0.64, SD 0.12); however, this domain improves substantially (mean 0.78, SD 0.14) when male and female sterilization (available in only 3 of the sample facilities) is excluded from consideration. Regarding the “current stock” domain (mean 0.71, SD 0.17), less than half of the facilities had female condoms at the time of the survey (mean 0.46, SD 0.50); two-thirds of the facilities had emergency contraceptive pills (mean 0.68, SD 0.47). Similarly, the stock-out score (mean 0.69, SD 0.30) was low; half of the facilities reported a stock-out of female condoms, and nearly a third reported stock-outs of emergency contraceptive pills, within the previous 3 months.

**TABLE 3.** Family Planning Readiness Domain Scores Among Study Facilities (N=68), Central Mozambique

	**Mean (SD)**
Overall family planning readiness score^a^	0.69 (0.20)
Provision and prescription readiness domain score	0.64 (0.12)
Facility provides or prescribes any of the following modern methods of family planning:	
Combined estrogen progesterone oral contraceptive pills	0.94 (0.24)
Progestin-only contraceptive pills	0.90 (0.31)
Combined estrogen progesterone injectable contraceptives	0.35 (0.48)
Progestin-only injectable contraceptives	0.72 (0.45)
Male condoms	0.87 (0.34)
Female condoms	0.57 (0.50)
Intrauterine contraceptive device	0.97 (0.17)
Implants	0.97 (0.17)
Emergency contraceptive pills	0.71 (0.46)
Male sterilization	0.00 (0.00)
Female sterilization	0.04 (0.21)
Guidelines and checklists readiness domain score	0.74 (0.38)
Facility has the following documents available at time of survey:	
National family planning guidelines	0.66 (0.48)
Family planning checklists and/or job aids	0.81 (0.40)
Trained staff readiness domain score	0.72 (0.45)
Facility family planning staff members received any family planning training in the last 2 years	0.72 (0.45)
Current stock readiness domain score	0.71 (0.17)
Facility has the following reproductive health medicines and commodities available at the time of survey^b^:	
Combined estrogen progesterone oral contraceptive pills	0.82 (0.38)
Progestin-only contraceptive pills	0.77 (0.42)
Combined estrogen progesterone injectable contraceptives	0.27 (0.45)
Progestin-only injectable contraceptives	0.71 (0.46)
Male condoms	0.72 (0.45)
Female condoms	0.46 (0.50)
Implants	0.94 (0.24)
Emergency contraceptive pills	0.68 (0.47)
Intrauterine contraceptive device	0.93 (0.26)
Stock-out readiness domain score	0.69 (0.30)
Facility had a stock-out in the past 3 months for each of the following^c^:	
Female condoms	0.50 (0.50)
Implants	0.90 (0.30)
Emergency contraceptive pills	0.67 (0.47)

a Overall family planning readiness and domain scores are averages of individual component indicators on a scale of 0 (lowest) to 1 (highest). All individual component indicators are binary from 0 (No) to 1 (Yes) unless otherwise specified.

b Never available or not available today (0); reported available but not seen (0.33); available but not valid (0.66); at least 1 available and valid (1).

c Facility stock-out register is not available, or the facility had a stock-out of any length of time during the previous 3 months, or the product is not provided or prescribed, or facility stock-out register is not filled in (0); facility has not had any stock-outs during the previous 3 months (1).

### Association Between Management Effectiveness and FP Facility Readiness

In the unadjusted analysis (Model A), higher overall management-effectiveness scores were independently associated with higher facility-readiness scores for FP services (Table 4). A 1-unit increase in the overall management effectiveness score was associated with a 7.15-fold increase in the odds of having a facility-readiness score above the median quantile (*P*<.001). When adjusting for facility type and distance from the provincial health directorate (Model C), we saw a significant association in the same direction, but the strength of the relationship weakened to a 4.88-fold increase (*P*=.001). Urban health centers and health centers adjoining hospitals were 2.10 and 4.26 times more likely to have a facility readiness score above the median quantile, respectively, but neither of these associations was statistically significant (*P*=.318, *P*=.399).

**TABLE 4.** Logistic Quantile Regression Model Estimates for Overall Family Planning Readiness, Central Mozambique

	**Model A** ^a^		**Model B** ^b^		**Model C** ^c^	
Variable	OR (95% CI)	*P* value	OR (95% CI)	*P* value	OR (95% CI)	*P* value
Overall management score	7.15 (3.02, 16.91)^d^	<.001^d^	4.70 (1.95, 11.30)^d^	<.001^d^	4.88 (1.86, 12.82)^d^	.001
Facility type						
Rural health center	-		reference		reference	
Urban health center	-		2.29 (0.65, 8.04)	.198	2.10 (0.49, 8.96)	.32
Adjoining hospital	-		4.09 (0.12, 138.12)	.432	4.26 (0.15, 123.40)	.40
Distance to DPS in 10 km	-		-		0.99 (0.95, 1.04)	.79
Years’ experience as manager	1.30 (1.03, 1.63)^d^	.025^d^	1.20 (1.02, 1.43)^d^	.0317^d^	1.21 (1.01, 1.45)^d^	.042^d^
Facility type						
Rural health center	-		reference		reference	
Urban health center	-		5.23 (1.18, 23.06)^d^	.029^d^	5.12 (0.98, 26.66)	.05
Adjoining hospital	-		4.38 (0.28, 67.42)	.289	4.42 (0.28, 70.88)	.29
Distance to DPS in 10 km	-		-		1.00 (0.93, 1.07)	.96
First-time facility manager	3.20 (1.21, 8.48)^d^	.019^d^	2.12 (0.93, 4.82)	.073	2.12 (0.89, 5.01)	.09
Facility type						
Rural health center	-		reference		reference	
Urban health center	-		3.82 (0.84, 17.39)	.083	3.84 (0.71, 20.79)	.12
Adjoining hospital	-		6.11 (0.08, 466.80)	.413	6.10 (0.08, 481.45)	.42
Distance to DPS in 10 km	-		-		1.00 (0.94, 1.06)	.99

Abbreviations: CI, confidence interval; DPS, provincial health directorate; OR, odds ratio.

a Unadjusted.

b Adjusted for facility type (rural center, urban center, center adjoining hospital).

c Adjusted for facility type (as above) and distance to DPS.

d Indicates statistical significance at *P*<.05.

Higher overall management-effectiveness scores were independently associated with higher facility-readiness scores for FP services.

### Association Between Management Experience and FP Facility Readiness

Our analysis of 2 additional explanatory variables related to management experience also had significant results. First, in our unadjusted model (Model A), each additional year of management experience was associated with a 1.30-fold increase in the odds of scoring above the median quantile overall FP readiness score (*P*=.025). This association maintained strength and significance even after adjusting for facility type and distance from the provincial health directorate. Adjusting for facility type only (Model B), urban health centers were 5.23 times more likely to have a readiness score above the median quantile (*P*=.029). Second, in our final model, which adjusted for facility type and distance from the provincial health directorate (Model C), results showed that being a first-time facility manager was associated with a 3.20-fold increase in the odds of having a readiness score above the median quantile (*P* =.019); however, the direction of this association is unexpected given that first-time managers presumably have less management experience.

## DISCUSSION

To our knowledge, this is the first study to quantify the association between management effectiveness and facility-level FP service readiness among PHC facilities in Mozambique. Findings from our model estimates showed a significant association between increased management capacity and higher levels of facility readiness for FP services. We also found that over two-thirds (n=47, 69.1%) of individuals were first-time managers, with three-quarters of them having less than 3 years of management experience.

Several important findings characterize the PHC management and FP service-readiness environment in central Mozambique. We found that only approximately one-quarter of the PHC facility managers in our sample received any kind of formal management training, which reflects the scarcity of investments in building management capacity in the health workforce in Mozambique. The lack of investment in management capacity may be attributable to the Ministry of Health’s focus instead on workforce expansion—from 1.87 health workers per 1,000 people in 2015 to the Sustainable Development Goal index threshold of 4.45.^26^ Deficits in management capacity may also be a consequence of the significant reductions in external financial support for the Mozambican government after the hidden debts scandal in 2016, which led to severe health sector spending cuts.^27^ The lack of trained managers may also indicate a reluctance to invest scarce resources in building management capacity among a workforce that experiences a high degree of turnover and migration to nongovernmental organizations, donor institutions, the private sector, and other countries.^28^ There is clear evidence that increasing the number of health workers can improve service delivery, but additional research is needed to investigate how management quality is associated with service readiness and health outcomes.^12^^,^^29^^,^^30^ This association is especially salient for PHC research, as management strength does not affect any one health service or outcome alone, but instead supports a broad range of PHC services and outcomes.

The lack of investment in management capacity may be attributable to the Ministry of Health’s focus instead on workforce expansion.

Furthermore, we found that facility managers spent over a third of their time on nonmanagerial tasks such as clinical duties, which may reduce their focus on management duties and ultimately contribute to reduced service readiness. This finding is consistent with previous research describing the shortage of health workers, specifically district management teams, and the implications this shortage has on management capacity.^9^

We found that many facility managers did not conduct staff performance reviews, and that performance reviews generally are not linked to staff salaries. This finding highlights an important gap in facility-level performance management that is well-documented in other studies included in a 2008 systematic review of the motivation and retention of health workers in developing countries.^31^ In the absence of routine staff performance management, facility managers are unable to identify areas for provider improvements that may directly translate to improved outputs and outcomes at reduced costs.^32^^,^^33^ Furthermore, the absence of performance reviews, as well as of compensation and incentives commensurate with performance, may be driving a lack of motivation in the public sector workforce and internal migration.^28^ A 2008 systematic review of health worker motivation and retention identified evidence of the positive impact of financial and nonfinancial incentives, such as career development and personal recognition, in improving PHC workforce motivation and retention.^31^

Similarly, the facilities included in our analysis also revealed significant gaps in the quality and frequency of external supervision visits, which is an essential management tactic to better integrate and improve service management at the facility level.^34^ A 2008 study on strengthening integrated PHC in Sofala province suggests that the quality and frequency of external supervision are linked to systems accountability and improvements in management-related health system deficiencies, highlighting the need for additional capacity at the provincial level to ensure meaningful and sustainable improvements at the district facility level.^9^

Regarding service readiness, higher management effectiveness scores were significantly associated with overall FP service readiness scores. This finding is consistent with similar research in Ghana and Ethiopia that found positive associations between a higher degree of facility-level management effectiveness and FP process outcomes.^13^^,^^14^ Our finding that urban facilities were significantly associated with higher levels of readiness for FP services differed from other recent evidence examining SARA FP data in 10 countries in sub-Saharan Africa.^35^ Whereas an assessment of FP service availability and readiness in 10 African countries found that rural facilities generally have more availability of contraceptives than urban facilities, the rural facilities in our sample generally had less availability.^35^ However, our finding is consistent with recent research on stock-outs of essential medicines, including contraceptive methods, in central Mozambique. In the study, each 10 km increase in distance from the facility to the district warehouse was associated with a 19% increase in the rate of stock-outs, and contraceptive stock-outs were common.^36^ Of note, the study found that routine provincial supervisory visits were associated with 4% lower stock-out rates.^36^ Similarly, previous qualitative research from Tanzania indicates that lack of routine supervisory visits may be related to shortages in medicine supply, as it is common practice for medications to be supplied during supervisory visits.^37^ This evidence may be related to our finding that a substantial proportion of PHC facilities did not consistently receive routine supervisory visits from provincial health managers. Future qualitative research could provide important context to the situation among the facilities in this study. Finally, we found that first-time facility managers were more likely to have a readiness score above the median quantile, which was unexpected. Additional research is needed to understand the determinants of this association.

### Limitations

Findings from our study have several limitations to consider when interpreting the results. First, this was a cross-sectional study that used secondary data to examine a statistical association between facility-level management effectiveness and FP-service readiness. Although we cannot attribute causality from this observational study using secondary data, the IDEAs survey is being repeated annually over 4 years to assess the impact of the IDEAs intervention on management effectiveness and measures of structural readiness. Second, the sample size is relatively small, contained a limited number of urban facilities compared to rural facilities, and represents only 4 out of 11 provinces in the country—all of which limit the generalizability and statistical power of the results. Future studies with larger sample sizes and more balance across urban and rural sites would increase statistical power and generalizability. Third, the 2018 SARA survey data set used in this analysis only contained data for 3 tracer indicators (female condoms, implants, and emergency contraceptive pills) in the “stock-out” domain, rather than for all 9 contraceptive methods evaluated in the “current stock” domain, including injectables and birth control pills. This discrepancy limited our ability to derive a more generalized stock-out score and, therefore, readiness across other service areas. Given that the most common method of modern contraception in Mozambique is injectables, understanding the stock-out history for this method is critical to fully understand FP service readiness.^15^^,^^38^ Finally, in measuring our outcome variable—facility readiness for FP services at the PHC facility level—we considered component indicators supported by the literature, but we also attributed equal importance to them. In reality, they may not be equally weighted, contributing to the low internal consistency of the variable. Further assessment of such psychometric properties is needed, which we could not perform given the small size of the sample and limited data availability. In general, the absence of consensus on the most appropriate PHC performance indicators and a common framework onto which they can be mapped out, particularly with respect to management effectiveness, poses a noteworthy challenge to comprehensively address this knowledge gap.^12^^,^^33^

## CONCLUSIONS

Our finding that higher levels of management effectiveness is independently associated with an increased likelihood of improved FP service readiness is an important contribution to the evidence base for management-related PHC implementation research. Additional data and research are needed to examine the drivers for facility-level and provincial-level variations in management effectiveness and service readiness for FP. Our findings also offer preliminary insights into gaps in management training experience and variations in management practices among PHC facility managers, as well as external supervisory practices, in central Mozambique.

These findings have important implications both within Mozambique and for the larger global health community. For Mozambique, these findings may motivate provincial-level policy makers to examine the drivers of variation in management practices, particularly for provincial-level supervisory support provided to district management teams. These results also illustrate the potential benefits of committing additional resources to developing facility-level management capacity and strengthening external supervisory support to improve the likelihood of improved FP service readiness and, ultimately, FP service delivery. At the global level, our findings contribute to an under-researched yet important body of literature on PHC management effectiveness and its role in service readiness in low- and middle-income countries. Future studies on this subject may benefit from the development of standardized indicators and instruments to measure management effectiveness, which could improve comparability and generalizability of similar research across heterogeneous settings and PHC more broadly.

## Supplementary Material

GHSP-D-21-00706-supplement1.pdf

GHSP-D-21-00706-supplement4.pdf

GHSP-D-21-00706-supplement3.pdf

GHSP-D-21-00706-supplement2.pdf

## Translation

**Em Português**

**Title**

Eficácia da Gestão dos Cuidados de Saúde Primários como Força Motriz da Prontidão de Serviço de Planeamento Familiar: Uma Análise Transversal no Centro de Moçambique

**Mensagens chave**

• Uma maior capacidade de gestão entre os gestores de unidades de atenção primária à saúde está associada a uma melhor prontidão dos serviços de planejamento familiar.

• Lacunas no desempenho de práticas de gestão são comuns, tanto nas unidades de cuidados de saúde primários bem como entre os respectivos supervisores provinciais externos.

• Entre os gestores de cuidados de saúde de atenção primária é comum haver deveres clínicos partilhados, e são generalizadas as lacunas no treino formal em gestão.

• Indicadores válidos, praticáveis, e sensíveis às práticas de gestão e prontidão de cuidados de saúde de atenção primária, podem ajudar os pesquisadores de saúde pública a melhorar a comparabilidade e generalização de pesquisa de implementação em atenção primária.

**Resumo**

**Introdução:** A fortaleza de gestão ao nível das unidades sanitárias é importante determinante da cobertura e qualidade dos cuidados de saúde de atenção primária; contudo, a relação entre gestão a nível da unidade sanitária e eficácia do sistema de saúde carece de suficiente base empírica. Descreve-se a associção entre efetividade de gestão e prontidão para prover serviços de planeamento familiar no centro de Moçambique.

**Métodos:** Uniram-se os dados da Avaliação de Disponibilidade e Prontidão de Serviços (SARA, sigla em Inglês) 2018 do Ministério de Saúde e os dados de uma segunda avaliação em 2018 contendo indicadores do módulo do Banco Mundial de gestão de prestação de seriviço. A análise centrou-se em 68 unidades de cuidados primários de saúde do sector público nas províncias de Manica, Sofala, Tete e Zambézia nas quais as duas avalições sobrepuseram-se. A regressão quantílica logística foi usada para modelar a associação entre a fortaleza de gestão e a prontidão de serviço de planejamento familiar.

**Resultados:** Dos 68 gestores de unidade sanitária, 69.1% (47) eram gestores pela primeira vez, e 26.5% (18) tiveram treino de gestão formal. Em média, os gestores indicaram que 63.6% do seu tempo de serviço era dispendido en responsabilidades de gestão, 63.2% dos trabalhadores haviam recebido uma avaliação de desempenho nos 12 meses precedentes a pesquisa, e 12.5% dos incentivos dos trabalhadores estava ligada à avaliação de performance. Ajustando para o tipo de unidade sanitária e distância à capital provincial, a efetividade de gestão e localização em zona urbana da unidade sanitária foram significativamente associados à alta prontidão para prestação de serviços de planeamento familiar.

**Conclusões:** Este estudo encontrou que a maior eficácia da gestão está independentemente associada a uma maior probabilidade de melhor prontidão para o serviço de planejamento familiar. Ademais, o estudo descreve as barreiras para a gestão eficaz dos serviços de atenção primária à saúde (APS), incluindo a falta de treinamento adequado para os gestores e atribuições partilhadas entre os gestores e clínicos, o que reduz o tempo disponível para as atividades de gestão. O fortalecimento das capacidades de gestão e o reforço das funções de gestão no nível da APS são necessários para melhorar a prontidão e os resultados do sistema de saúde, o que é essencial para alcançar os ODS globais e as metas de cobertura universal de saúde.

**Peer Reviewed**
