# Supplementary material for: Primary Health Care Management Effectiveness as a Driver of Family Planning Service Readiness: A Cross-Sectional Analysis in Central Mozambique
Source: Glob Health Sci Pract. 2022 Sep 15;10(Suppl 1):e2100706. doi: 10.9745/GHSP-D-21-00706 (PMC9476484; doi:10.9745/GHSP-D-21-00706)
Supplement: GHSP-D-21-00706-supplement1.pdf [file GHSP-D-21-00706-supplement1.pdf]

Supplement to: Pope S, Augusto O, Fernandes Q, et al. Primary health care management effectiveness as a driver of family planning service readiness: a cross-sectional analysis in central Mozambique. *Glob Health Sci Pract*. 2022;10(Suppl 1):e2100706. https://doi.org/10.9745/GHSP-D-21-00706

Supplement 1. Management effectiveness survey questions for health facility managers

The sampled health facility managers were asked to respond to the following 15 questions related to health facility management practices.

Management Practices

1. On a typical day, how much time do you spend on each activity (in hours)?
    a. Supervise patient flow (e.g. patient admission, transfer discharge, screening)
    b. Supervise clinical staff (e.g. check absences, check for treatment by nurses and physicians)
    c. Distribution of tasks
    d. Reporting / completion of bulletins / administrative activities
    e. Check equipment and availability of medications
    f. Treating patients
    g. Manage relationships with health professionals, community, health unit committee, donors, and government
    h. Other
2. Do you keep records of staff attendance?
    a. Yes
    b. No
3. In the past 12 months, did you (the person in charge of the health facility) have an individual meeting with each employee to review/evaluate their performance?
    a. Yes
    b. No
4. Are performance reviews related to salaries and incentives that staff members receive?
    a. Yes
    b. No
5. In the past 12 months, how many times did this health facility request medications?

External Supervision

1. During the past six months, how many supervision or technical assistance visits have you received from representatives of Provincial Health Directorate (DPS) or SDSMAS?
2. During the last visit, did the external supervisor use a control list?
    a. Yes
    b. No
3. During the last visit, did the external supervisor observe consultations?
    a. Yes
    b. No
4. During the last visit, did the external supervisor observe staff attendance logs?
    a. Yes
    b. No
5. During the last visit, did the external supervisor observe stock of medications?
    a. Yes
    b. No
6. During the last visit, did the external supervisor observe financial registries?

   a. Yes
   b. No

7. During the last visit, did the external supervisor write feedback in the supervisory log of this health facility?
   a. Yes
   b. No

Community Health Committee

1. In the past 12 months, how many times did the Community Health Committee meet?
2. Does this health facility have a formal mechanism to collect patient opinions (surveys, suggestion box, other)
   a. Yes
   b. No
3. In the past six months, were management changes made as a result of patient opinions?
   a. Yes
   b. No
