# Supplementary material for: Primary Health Care Management Effectiveness as a Driver of Family Planning Service Readiness: A Cross-Sectional Analysis in Central Mozambique
Source: Glob Health Sci Pract. 2022 Sep 15;10(Suppl 1):e2100706. doi: 10.9745/GHSP-D-21-00706 (PMC9476484; doi:10.9745/GHSP-D-21-00706)
Supplement: GHSP-D-21-00706-supplement4.pdf [file GHSP-D-21-00706-supplement4.pdf]

Supplement to: Pope S, Augusto O, Fernandes Q, et al. Primary health care management effectiveness as a driver of family planning service readiness: a cross-sectional analysis in central Mozambique. *Glob Health Sci Pract*. 2022;10(Suppl 1):e2100706. https://doi.org/10.9745/GHSP-D-21-00706

Supplement 4. Family planning facility readiness component indicators based on the Service Availability and Readiness Assessment framework

The following family planning facility readiness component indicators are based on the Service Availability and Readiness Assessment (SARA) survey.

	**Questions to health facility:**		
**Family planning Provision**		**0**	**1**
	1. Does this facility provide or prescribe any of the following modern methods of family planning?		
	a. Combined estrogen progesterone oral contraceptive pills	No	Yes
	b. Progestin-only contraceptive pills	No	Yes
	c. Combined estrogen progesterone injectable contraceptives	No	Yes
	d. Progestin-only injectable contraceptives	No	Yes
	e. Male condoms	No	Yes
	f. Female condoms	No	Yes
	g. Intrauterine contraceptive device (IUCD)	No	Yes
	h. Implants	No	Yes
	i. Emergency contraceptive pills	No	Yes
	j. Male sterilization	No	Yes
	k. Female sterilization	No	Yes
	**Provision readiness score:**	**___________ / 11**	
**Guidelines & Checklists**		**0**	**1**
	1. Are any of the following documents available in the facility today?		
	a. National family planning guidelines	No	Yes
	b. Family planning checklists and/or job-aids	No	Yes
	**Guideline & Check-list readiness score:**	**___________ / 2**	
**Training**		**0**	**1**
	1. Have you or any provider(s) of family planning services received any family planning training in the last two years?	No	Yes
	**Training readiness score:**	**___________ / 1**	

Supplement to: Pope S, Augusto O, Fernandes Q, et al. Primary health care management effectiveness as a driver of family planning service readiness: a cross-sectional analysis in central Mozambique. *Glob Health Sci Pract*. 2022;10(Suppl 1):e2100706. https://doi.org/10.9745/GHSP-D-21-00706

**Stock Readiness**		
	1. Are any of the following reproductive health medicines and commodities available in this service site today?<br>**Score conversion: 1 = At least one valid | 0.66 = Available but not valid | 0.33 = Available but not seen | 0 = Not available today or Never available**	Score:
	a. Combined estrogen progesterone oral contraceptive pills	
	b. Progestin-only contraceptive pills	
	c. Combined estrogen progesterone injectable contraceptives	
	d. Progestin-only injectable contraceptives	
	e. Male condoms	
	f. Female condoms	
	g. Implant	
	h. Emergency contraceptive pills	
	i. Intrauterine contraceptive device (IUCD)	
	**Stock readiness score:**	**___________ / 9**
**Stockout**		**Score**
	1. For each of the following items, please check in the facility records if there has been a stock-out in the past three months:<br>**Score conversion: 1 = No stockouts | 0 = At least one stockout for any period of time OR Stock registry not available OR Stock registry not filled in OR Product not provided or prescribed**	Score:
	a. Female condoms	
	b. Implants	
	c. Emergency contraceptive pills	
	**Stockout readiness score:**	**___________ / 3**
	**TOTAL FAMILY PLANNING READINESS SCORE:**	
