# Supplementary material for: Primary Health Care Management Effectiveness as a Driver of Family Planning Service Readiness: A Cross-Sectional Analysis in Central Mozambique
Source: Glob Health Sci Pract. 2022 Sep 15;10(Suppl 1):e2100706. doi: 10.9745/GHSP-D-21-00706 (PMC9476484; doi:10.9745/GHSP-D-21-00706)
Supplement: GHSP-D-21-00706-supplement3.pdf [file GHSP-D-21-00706-supplement3.pdf]

Supplement to: Pope S, Augusto O, Fernandes Q, et al. Primary health care management effectiveness as a driver of family planning service readiness: a cross-sectional analysis in central Mozambique. *Glob Health Sci Pract*. 2022;10(Suppl 1):e2100706. https://doi.org/10.9745/GHSP-D-21-00706

Supplement 3. Service Delivery Indicator management indicators

The following management indicators are based on the Service Delivery Indicator survey.

	**Questions to facility manager:**			
**Management Practices**		**0**		**1**
	1. Time spent for managerial activities on a typical day:<br>a. Supervise patient flow (e.g., patient admission, transfers, discharges, screening);<br>b. Supervise clinical staff (e.g., check absences, check for treatment by nurses and physicians);<br>c. Distribution of tasks;<br>d. Reporting, completion of bulletins, administrative activities;<br>e. Check equipment and availability of medications;<br>Manage relationships with health professionals, community, health unity committee, donors, and government	Proportion of average managerial time spent:		
	2. Do you keep records of staff attendance?	Do not know / No		Yes
	3. In the past 12 months, did you have an individual meeting with each employee to review/evaluate their performance?	Do not know / No		Yes
	4. Are performance reviews related to salaries and incentives that staff members receive?	Do not know / No		Yes
	5. In the past 12 months, how many times did this health facility request medications?<br>**Score conversion: 1 = 12 | 0 = < 12**	Proportion of average number of medication requests:		
	**Management Practices score:**	**___________ / 5**		
**External Supervision**		**0**	**0.5**	**1**
	1. During the past six months, how many supervision or technical assistance visits have you received from representatives of Provincial Health Directorate (DPS) or SDSMAS?<br>**Score conversion: >3 = 0 | >= 3 and < 5 = 0.5 | >= 5 = 1**	>3 visits	>= 3 and <5 visits	>= 5 visits
	2. During the last visit, did the external supervisor use a control list?	Do not know / No		Yes

Supplement to: Pope S, Augusto O, Fernandes Q, et al. Primary health care management effectiveness as a driver of family planning service readiness: a cross-sectional analysis in central Mozambique. *Glob Health Sci Pract*. 2022;10(Suppl 1):e2100706. https://doi.org/10.9745/GHSP-D-21-00706

	3. During the last visit, did the external supervisor observe consultations?	Do not know / No		Yes
	4. During the last visit, did the external supervisor observe staff attendance logs?	Do not know / No		Yes
	5. During the last visit, did the external supervisor observe stock of medications?	Do not know / No		Yes
	6. During the last visit, did the external supervisor observe financial registries?	Do not know / No		Yes
	7. During the last visit, did the external supervisor write feedback in the supervisory log of this health facility?	Do not know / No	Yes, but did not see it	Yes, and read it
	**External Supervision score:**	**__________ / 7**		
**Community Engagement**		**0**		**1**
	1. In the past 12 months, how many times did the Community Health Committee meet?<br>**Score Conversion: 1 = >= 12 | 0.5 = >= 6 & < 12 | 0.25 = >= 3 & < 6 | 0 = < 3**	Score:		
	2. Does this health facility have a formal mechanism to collect patient opinions? (surveys, suggestion box, other)	Do not know / No		Yes
	3. In the past six months, were management changes made as a result of patient opinions?	Do not know / No		Yes
	**Community Engagement score:**	**__________ / 3**		
	**TOTAL MANAGEMENT SOCRE:**			
