# Supplementary material for: Primary Health Care Management Effectiveness as a Driver of Family Planning Service Readiness: A Cross-Sectional Analysis in Central Mozambique
Source: Glob Health Sci Pract. 2022 Sep 15;10(Suppl 1):e2100706. doi: 10.9745/GHSP-D-21-00706 (PMC9476484; doi:10.9745/GHSP-D-21-00706)
Supplement: GHSP-D-21-00706-supplement2.pdf [file GHSP-D-21-00706-supplement2.pdf]

Supplement to: Pope S, Augusto O, Fernandes Q, et al. Primary health care management effectiveness as a driver of family planning service readiness: a cross-sectional analysis in central Mozambique. *Glob Health Sci Pract*. 2022;10(Suppl 1):e2100706. https://doi.org/10.9745/GHSP-D-21-00706

Supplement 2. Family planning service readiness questions from Mozambique's 2018 Service Availability and Readiness Assessment (SARA) survey

The following five questions are from Mozambique's 2018 SARA survey within the family planning service readiness module.

1. Does this facility provide or prescribe any of the following modern methods of family planning:
   a. Combined estrogen progesterone oral contraceptive pills
   b. Progestin-only contraceptive pills
   c. Combined estrogen progesterone injectable contraceptives
   d. Progestin-only injectable contraceptives
   e. Male condoms
   f. Female condoms
   g. Intrauterine contraceptive device (IUCD)
   h. Implants
   i. Emergency contraceptive pills
   j. Male sterilization
   k. Female sterilization
2. Please tell me if the following documents are available in the facility today:
   a. National family planning guidelines
   b. Family planning checklists and/or job-aids
3. Have you or any provider(s) of family planning services:
   a. Received any family planning training in the last two years?
4. Are any of the following reproductive health medicines and commodities available in this service site today?
   a. Combined estrogen progesterone oral contraceptive pills
   b. Progestin-only contraceptive pills
   c. Combined estrogen progesterone injectable contraceptives
   d. Progestin-only injectable contraceptives
   e. Male condoms
   f. Female condoms
   g. Implant
   h. Emergency contraceptive pills
   i. Intrauterine contraceptive device (IUCD)
5. For each of the following items, please check in the facility records if there has been a stock-out in the past three months:
   a. Female condoms
   b. Implant
   c. Emergency contraceptive pills
